# Hashimoto’s thyroiditis impairs embryo implantation by compromising endometrial morphology and receptivity markers in euthyroid mice

**DOI:** 10.1186/s12958-019-0526-3

**Published:** 2019-11-15

**Authors:** Zhangbi Wu, Yaojun Cai, Qin Xia, Tiantian Liu, Hao Yang, Fen Wang, Nan Wang, Zhen Yu, Chunying Yin, Qunan Wang, Defa Zhu

**Affiliations:** 1 Department of Geriatric Endocrinology, the First Affiliated Hospital of Anhui Medical University, Hefei, 230032 China; 2 Department of Endocrinology, the First Affiliated Hospital of Nanchang University, Nanchang, 330006 China; 3 Anhui Province Key Laboratory of Reproductive Health and Genetics, Anhui Medical University, Hefei, 230032 China; 4 Center for Integrative Imaging, Hefei National Laboratory for Physical Sciences at Microscale, University of Science and Technology of China, Hefei, 230027 China; 5 Department of Toxicology, School of Public Health, Anhui Medical University, Hefei, 230032 China

**Keywords:** Hashimoto’s thyroiditis, Embryo implantation, Endometrial receptivity, Pinopodes, Receptivity markers

## Abstract

**Background:**

Although thyroid dysfunction caused by Hashimoto’s thyroiditis (HT) is believed to be related to implantation failure due to the underdevelopment of the receptive uterus, it is unknown whether HT itself, even in the euthyroid state, impairs embryo implantation associated with endometrial receptivity defects. To address whether HT itself can affect endometrial receptivity accompanied by implantation alterations, a euthyroid HT model was established in mice.

**Methods:**

Female NOD mice were immunized twice with thyroglobulin and adjuvant to induce the experimental HT model. Four weeks after the second treatment, the mice were normally mated, and pregnant ones were sacrificed in implantation window for thyroid-related parameter and steroid hormones measurements by electrochemiluminescence immunoassay and enzyme-linked immunosorbent assay and implantation site number calculation by uptake of Chicago Blue dye. In addition, certain morphological features of endometrial receptivity were observed by hematoxylin-eosin staining and scanning electron microscopy, and the expression of other receptivity markers were analyzed by immunohistochemistry, RT-qPCR or Western Blot.

**Results:**

HT mice displayed intrathyroidal monocyte infiltration and elevated serum thyroid autoantibody levels without thyroid dysfunction, defined as euthyroid HT in humans. Euthyroid HT resulted in implantation failure, fewer pinopodes, retarded pinopode maturation, and inhibited expression of receptivity markers: estrogen receptor α (ERα), integrin β3, leukemia inhibitory factor (LIF), and cell adhesion molecule-1 (ICAM-1). Interestingly, despite this compromised endometrial receptivity response, no statistical differences in serum estradiol or progesterone level between groups were found.

**Conclusions:**

These findings are the first to indicate that HT induces a nonreceptive endometrial milieu in the euthyroid state, which may underlie the detrimental effects of HT itself on embryo implantation.

## Introduction

Hashimoto thyroiditis (HT) is the most frequent form of autoimmune thyroid disease (AITD), affecting up to 5% of the general population, predominantly childbearing-age women [1–3]. Hashimoto thyroiditis, which is characterized by enlarged thyroid gland, marked intrathyroidal monocyte infiltration, and elevated serum antithyroid autoantibody (ATA) concentrations, including anti-thyroglobulin antibody (Tg-Ab) and anti-thyroid peroxidase antibody (TPO-Ab) [4], is the most frequent underlying factor leading to hypothyroidism [5]. Hypothyroidism has been frequently linked to embryo implantation, since alterations of the highly regulated local activity of thyroid-stimulating hormone (TSH) and thyroid hormones (TH) directly interfere with embryo attachment and early implantation [6, 7]. Nevertheless, approximately 79.3% of HT patients display a euthyroid state at diagnosis and may retain normal thyroid function for many years [8, 9]. In recent years, the association between euthyroid HT and pregnancy loss has drawn attention [10, 11]. Data from the literature suggest that the leading single cause of pregnancy failure is embryo implantation error, which can occur at a rate of up to 78% in humans [12]. A study clearly established that in the absence of thyroid function disorders, thyroid autoimmunity with rising serum TG-Ab and TPO-Ab levels is associated with repeated implantation failure [13]. Taken together, these data strongly suggest that HT itself, independent of thyroid hormone level, may be a primary factor in embryo implantation failure.

Blastocyst or embryo implantation is critical for the establishment of pregnancy and occurs only for a limited time, defined as the “window of implantation (WOI),” between days 6 and 12 postfertilization in humans and between embryonic days 3.5 and 4.5 postcoitus in mouse [14]. A prerequisite for successful embryo implantation is a synchronized dialogue between the competent blastocyst and the receptive uterus [14]. Hashimoto thyroiditis, the most common autoimmune disease, is frequently encountered with other autoimmunological diseases, such as type 1 diabetes [15]. The illness has also been found to present a comorbidity with impaired implantation associated with certain morphological and molecular features that change uterine receptivity [16]. A receptive endometrium is one of the core factors for successful embryo implantation. The generation of the receptive uterus is characterized by the development of certain specific transformational changes at the molecular levels of the endometrium stromal cells and epithelium that occur only during the WOI. To allow implantation, morphological and biochemical reprogramming of the endometrium, called decidualization, is needed [17]. One characteristic change on the apical surface of the luminal epithelium in the mammalian endometrium in preparation for implantation is the formation of pinopodes [18], spherical protrusions of the epithelial plasma membrane into the lumen, which are classic morphological biomarkers of endometrial receptivity favoring implantation [19, 20]. Many patients who have infertility due to implantation defects fail to produce pinopodes [21]. The appearance of pinopodes is consistent with the expression of other markers of endometrial receptivity. The steroid hormones estradiol (E2) and progesterone (P), which exert function through their respective nuclear receptors, the estrogen receptor (mainly ERα but not ERβ) [22, 23] and the progesterone receptor (PR), are primarily responsible for the establishment of endometrial receptivity [24, 25]. Several molecular markers are involved in endometrial receptivity, including integrin β3, leukemia inhibitory factor (LIF), and cell adhesion molecule-1 (ICAM-1), which are crucial to embryo implantation [26, 27]. Given the significant implantation-favoring effects of pinopodes, steroid hormones and their receptors, integrin β3, LIF, and ICAM-1 on endometrial receptivity, we hypothesize that these events provide uterine factors for compromised embryo implantation in the context of euthyroid HT.

To test this hypothesis, this study built a classical HT mouse model [28] in which female NOD mice were actively immunized with porcine thyroglobulin (pTg) and investigated whether HT itself was able to affect the morphology of the endometrium and the molecular expression of endometrial receptivity-related genes accompanied by compromised embryo implantation in implantation window.

## Materials and methods

### Reagents and chemicals

Porcine thyroglobulin (pTg), complete Freund’s adjuvant (CFA) and incomplete Freund’s adjuvant (IFA) were from Sigma Chemical Co. (St. Louis, MO, USA). The TSH ELISA kit was from Cloud-Clone Corp. (Wuhan, Hubei, China). The E2 and P ELISA kits were from Cusabio Biotech Co., Ltd. (Wuhan, Hubei, China). The SPlink Detection kit was from ZSGB-Bio (Beijing, China). Estrogen receptor α, integrin β3 and GAPDH antibodies were from Abcam (Cambridge, MA, USA). Progesterone receptor, LIF, ICAM-1 antibodies were from Bioss, Inc. (Beijing, China). TRI reagent was from Molecular Research Center, Inc. (Cincinnati, OH, USA). Ribonuclease-free deoxyribonuclease (RNase-free DNase) and real-time reverse transcription (RT) kits were from Promega Corporation (Madison, WI, USA). Light Cycler® 480 SYBR Green I Master Mix was from Roche Diagnostics GmbH (Basel, Switzerland). All other reagents were purchased from Sigma or as indicated in the Methods.

### Animals

NOD mice (4 weeks old, female mice: 10~13 g; male mice: 12~16 g) were purchased from Nanjing Biomedical Research Institute of Nanjing University (Permit Number: 15–0001). After 7 days of quarantining, all mice were kept in specific pathogen-free conditions with ad libitum access to water and food in the Laboratory Animal Center of Anhui Medical University (Permit Number: 17–006). All procedures on animals were performed in accordance with the guidelines set by the Center for Laboratory Animal Sciences and the Association of Laboratory Animal Sciences at Anhui Medical University.

### Immunization and experimental design

After a week’s acclimation period, fifty-six female NOD mice were randomly divided into the control group (CON group, *n* = 28) and HT group (HT group, n = 28). Porcine thyroglobulin (25 μg) in sterile phosphate-buffered saline (PBS) was emulsified in 100 μl CFA and injected intradermally into the tail of the HT group. The mice were given a booster dose, except that pTg was emulsified in IFA 14 days later. Meanwhile, the controls were injected subcutaneously with the same volume of PBS without pTg in the emulsion. Four weeks after the repeated immunization, two immunized female mice were mated to one untreated male NOD mouse overnight, and the morning when a vaginal mucus plug was seen, considered the sign of successful coitus, was designated as embryonic day 0.5 (Day E0.5). In the morning (08:30–10:30 am) on Day E4.5, mice were bled and killed under deep anesthesia. Furthermore, 10 mice were selected from each group, and 0.1 ml 1% Chicago Blue dye in 0.9% NaCl was injected into the tail vein of each mouse, and then the mice were sacrificed 5 min later to observe the implantation sites demarcated by distinct blue nodules in uterus. A vaginal mucus plug plus the appearance of viable implant sites indicated successful pregnancy. Blood samples after 4–6 h of quiescence were centrifuged at 4000×g for 10 min to measure parameters in serum. Thyroid tissues were used for hematoxylin and eosin staining. The uteri from each group were carefully dissected, and pieces were allocated for subsequent assays: histopathology (3/group); scanning electron microscopy (3/group); endometrial homogenate parameters (6 left uterine horn/group); RT-PCR (6 right uterine horn/group); and western blot (6/group).

### Electrochemiluminescence immunoassay (ECLIA)

All serum and tissue samples were preserved at − 80 °C until use. In addition, endometrial tissues were homogenized in 10 μl/mg PBS, and then the supernatants were collected by centrifugation at 15,000×g for 15 min at 4 °C. The concentrations of free triiodothyronine (FT3), free tetraiodothyronine (FT4), TPO-Ab and Tg-Ab in serum and endometrial homogenate supernatant were assayed by electrochemiluminescence immunoassay (ECLIA) using a Cobas e411 clinical chemistry analyzer (Roche, Mannheim, Germany). Free triiodothyronine, FT4, TPO-Ab and Tg-Ab ECLIA kits were purchased from Roche Applied Science. The procedures for ECLIA were as described in detail elsewhere [29]. The results were determined via a calibration curve that was instrument-specifically generated by 2-point calibration and a master curve provided via the reagent barcode. Data are expressed as international units picomolar per gram hormone and per milligram protein of endometrial tissue. All samples were run in duplicate and the average was used as the final analysis value for each sample. Coefficients of variation for the assays of these thyroid profiles ranged from 7.38 to 14.22%.

### Enzyme-linked immunosorbent assay (ELISA)

The remaining serum samples were thawed to room temperature (18–25 °C) for TSH, E2 and P quantification in each month using their respective ELISA kits according to the manufacturer’s instructions. For the optical density (OD) value, the absorbance of the color in the plates was measured at 450 nm by a BioTek reader (Biotek Winooski, Vermont, USA). Data are expressed as picograms or nanograms per milliliter hormone of serum. All samples were run in duplicate and the average was used as the final analysis value for each sample. Coefficients of variation for the assays of the steroid hormones and TSH ranged from 7.24 to 9.84%.

### Hematoxylin and eosin (HE) staining and immunohistochemistry (IHC)

Freshly collected thyroid glands and uteri were fixed in 4% paraformaldehyde for 24 h on a shaker and then embedded in paraffin wax. From each paraffin-embedded tissue, coronal slices (3 μm thick) were serially sectioned. Hematoxylin and eosin (HE)-stained thyroid slices were quantified for thyroid mononuclear cell infiltration area in accordance with a previous study [30]: 0 = no infiltration; 1 = one or two follicular interstitia accumulated by inflammatory cells; 2 = one or two inflammatory cell lesions reaching follicular size; 3 = 10–40% inflammatory cell infiltration; 4 = more than 40% inflammatory cell infiltration. In addition, HE staining of the endometrium was analyzed for morphological observation using an Olympus DP80 microscope (Olympus, Tokyo, Japan). In each uterus, at least 3 noncontiguous sections were randomly selected to calculate the number of glands (40× magnification).

Immunohistochemistry (IHC) was performed using the SPlink Detection kit. Five-micrometer-thick uterus sections were mounted onto slides, deparaffinized and rehydrated through xylene and a graded alcohol series. After each step, the sections were rinsed 3 times with PBS (3 min each). After quenching endogenous peroxidase activity with 3% hydrogen peroxide for 10 min, antigen retrieval was performed by steaming the sections in 0.01 M citrate buffer (pH 6.0) for 20 min. Nonspecific binding sites were blocked with 5% normal goat serum for 30 min before the specific primary antibodies against ERα (ab96867, 1:250) and PR (bs23376R, 1:500), overnight at 4 °C. Slides were incubated for 30 min with biotinylated goat anti-rabbit IgG followed by 45 min incubation with horseradish peroxidase-labeled avidin-biotin complex. Immunostaining was developed by the application of diaminobenzidine. Slides were counterstained with hematoxylin, dehydrated, and mounted using mounting medium.

### Scanning electron microscopy (SEM)

To assess pinopode morphology, three uteri on day E5 in each group were cut open longitudinally to expose the uterine luminal epithelium, followed by gently rinsing the sample surface with PBS and rapid fixation in 2.5% glutaraldehyde. The fixed samples were rinsed 3 times (15 min each) in 0.1 M PBS, postfixed in 1% osmium tetroxide for 90 min without light, and further rinsed as before. Then, the 3-mm^2^ tissue blocks were dehydrated through a graded alcohol series (30, 50, 70, 80, 90, 95, 100%) and 100% acetone 3 times (10 min each), dried with liquid carbon dioxide in a critical point dryer (Quorum K850, UK), and coated with palladium gold using an ion sputter instrument (IXRF MSP-2S, USA) for 30 s. Scanning photomicrographs were acquired from three randomly selected surface fields of endometrial epithelium per sample to analyze the effect of HT on the pinopodes by scanning electron microscopy (Gemini SEM 500, Zeiss, Germany). The number of pinopodes per microscopic field was recorded (5000× magnification).

### Reverse transcription-quantitative polymerase chain reaction (RT-PCR)

Total RNA from endometrial tissues was extracted and purified using TRIzol reagent according to the manufacturer’s protocol. The concentration of RNA was determined by a NanoDrop2000 spectrophotometer (Thermo Fisher Scientific, MA, USA) and the integrity was detected by electrophoresis on agarose gels. Two micrograms of the total RNA for each sample was treated with RNase-free DNase at 37 °C for 30 min to remove any DNA contamination and then denatured with stop solution at 65 °C for 10 min. For first-strand complementary DNA (cDNA) synthesis, the RNA was then used as a template in a 20-μl RT reaction mixture containing 0.5 μl 50 U/ml ribonuclease inhibitor, 1 μl 0.5 mg/ml oligo (dT15), 2 μl 10 mM deoxynucleotide triphosphate (dNTP) mix, 0.75 μl of 200 U/μl of AMV reverse transcriptase, 2 μl 10× reverse transcription buffer, and 2.75 μl RNase-free water. Reactions were performed at 42 °C for 1 h and then 95 °C for 5 min. The final PCR mixture contained 10 μl Light Cycler® 480 SYBR Green I Master Mix (04887352001), 1 μl of cDNA, 2 μl 10 nM sense and antisense primers, and sterile water to 20 μl. Gene-specific primers were listed in Table 1. The PCR reactions were performed at 95 °C for 10 min, followed by 40 cycles of 95 °C for 15 s, 60 °C for 1 min, and 72 °C for 30 s. All reactions were performed in triplicate. The comparative cycle threshold method was used to determine the amount of target, normalized to an endogenous reference (β-actin), and relative to a calibrator (2^-△△CT^) [31] using the Light Cycler 480 software (version 1.5.0, Roche). The stability of the β-actin was verified by amplification and dissolution curves. Coefficients of variation for the assays of β-actin cycle threshold was lower 5%.

**Table 1:** Primers for Real-Time RT-PCR

Genes	Sequence	Sizes (bp)
β-actin	Forward: 5′-GTGACGTTGACATCCGTAAAGA-3’	287
	Reverse: 5′-GTAACAGTCCGCCTAGAAGCAC-3’	
Integrinβ3	Forward: 5′-GAGTGCTCTGAGGAGGATTACCG-3’	262
	Reverse: 5′-TGCAGTAGTAGCCAGTCCAGTCC-3’	
LIF	Forward: 5′-AACTGGCACAGCTCAATGGC-3’	198
	Reverse: 5′-TCAGGGAGGCGCTCAGGTAT-3’	
ICAM-1	Forward: 5′-CATCACCGTGTATTCGTTTCCG-3’	166
	Reverse: 5′-TGGCTGGCGGCTCAGTATCT-3’	

### Western blot

For nuclear protein extraction from the endometrium, tissue was homogenized on ice in buffer A [1 mM EDTA, 150 mM NaCl, 0.6% NP-40, 10 mM HEPES (pH 7.9), and 0.5 mM phenylmethylsulfonyl fluoride (PMSF)]. Then, the nuclear pellet was homogenized on ice for 1 h in buffer B [20 mM HEPES (pH 7.9), 0.5 mM dithiothreitol, 1.2 mM MgCl2, 25% glycerol, 0.2 mM EDTA, 420 mM NaCl, 0.5 mM PMSF and 1% protease inhibitor cocktail (P8340, Sigma)]. In addition, endometrial total protein was extracted on ice with tissue lysis buffer [1% Triton X-100, 1 mM EDTA, 50 mM Tris-HCl (pH 7.4), 1% sodium deoxycholate, 150 mM NaCl, 0.1% sodium dodecylsulfate (SDS)] and 1 mM PMSF in a glass homogenizer. Lysate were centrifuged at 15,000×g for 15 min at 4 °C to remove solid debris. The protein concentration was determined using the bicinchoninic acid (BCA) Protein Assay Kit (PA115, TianGen Biotech Co., Ltd., China) according to the manufacturer’s instructions. To perform immunoblotting, the same amount of protein for each sample was separated by SDS-PAGE and transferred to a polyvinylidene fluoride membrane. Nonspecific binding sites on the membranes were blocked in 5% skim milk in Dulbecco’s PBS (DPBS) for 1.5 h and incubated overnight at 4 °C with specific primary antibodies against ERα (ab96867; 1:1000), PR (bs23376R; 1:1000), integrin β3 (ab210515; 1:1000), LIF (bs1058R, 1:500), ICAM-1 (bs4617R; 1:1000), and GAPDH (ab8245; 1:4000). After 3 washes in DPBS containing 0.05% Tween-20 for 10 min each, membranes were incubated with goat anti-rabbit IgG (1:80,000) as the secondary antibody for 2 h. Protein bands were visualized by an enhanced chemiluminescence (ECL) detection kit (Thermo Fisher Scientific, Inc., Waltham, Ma, USA), and images were obtained using a Fine-do X6 visualizer (Tanon Science and Technology Co., Shanghai, China). GAPDH was used as an internal control.

### Statistical analysis

All data are presented as the mean ± SEM unless otherwise noted. The unpaired two-tailed Student’s t-test was performed to compare the variables between groups. The prevalence of pregnancy was assessed by the Fisher exact test. Implantation sites and thyroiditis score were compared by the Mann-Whitney test. All graphs were made using GraphPad Prism software version 7.0 (GraphPad Software, Inc., CA, USA). The western blots and immunohistochemistry slides were scanned, and the abundance was assessed quantitatively using Image-Pro Plus (Media Cybernetics, Inc., MD, USA). All quantitative data were analyzed using SPSS version 16.0 (IBM, Armonk, New York, USA). *p*-values < 0.05 were considered statistically significant.

## Results

### Building a euthyroid HT mouse model on Day E4.5

**Fig. 1:**
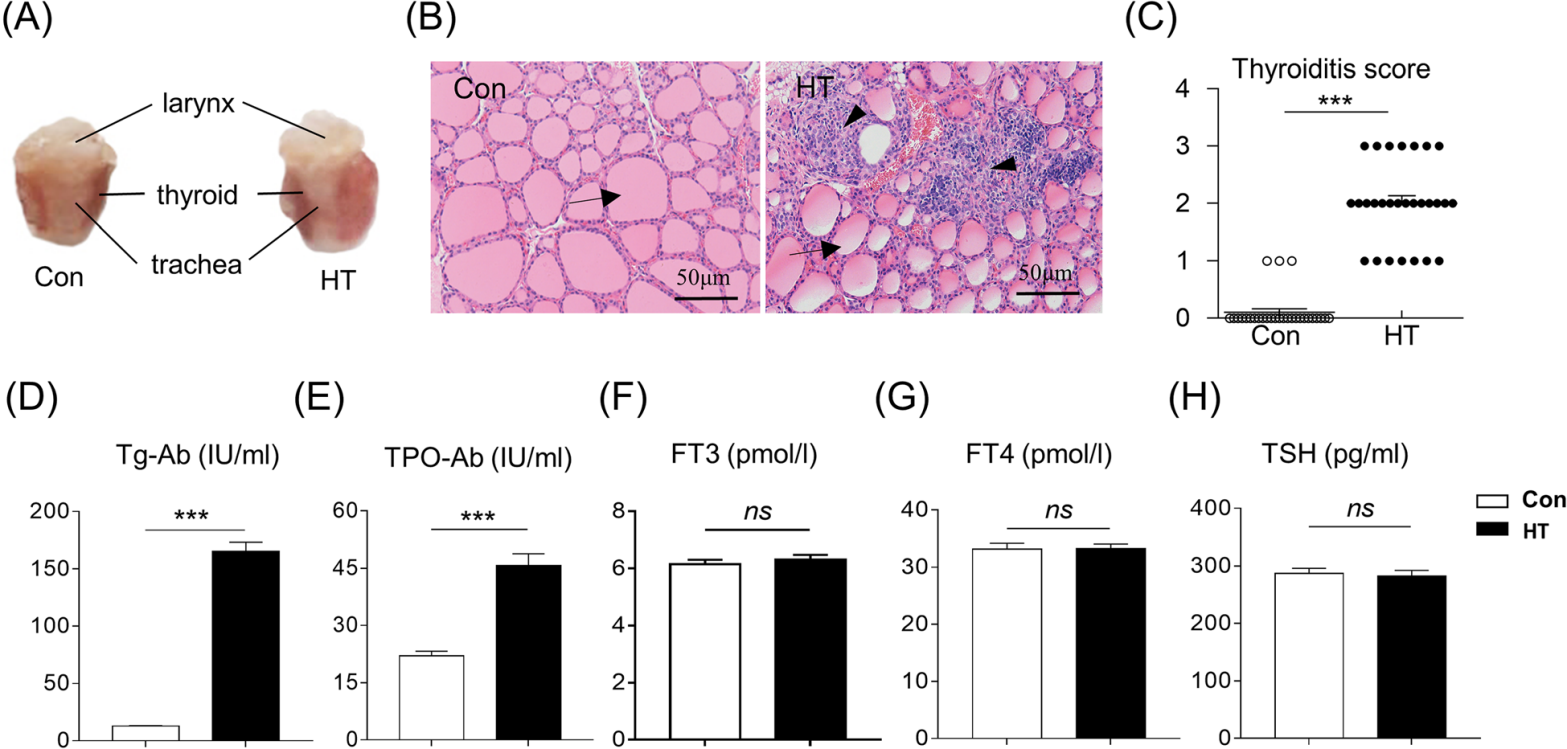
Building a euthyroid HT mouse model on Day E4.5. **a** Representative macroscopic images of thyroid glands from Con mice and HT mice. **b** Histology of the thyroid detected by HE staining at magnification × 200. Arrow: thyroid follicle; Arrowhead: infiltrated monocytes. **c-e** Levels of thyroiditis-related parameters. **c** Quantitation of the degree of monocyte infiltration in thyroids (Mann-Whitney test), **d** Serum anti-Tg, **e** Serum anti-TPO. **f-h** Serum levels of thyroid function-related parameters. **f** FT3, **g** FT4, **h** TSH. Data are presented as the mean ± SEM, *n* = 28; ns, not significant; ^***^p < 0.001, vs. Con

As depicted in Fig. 1a, mice immunized with Tg displayed pronounced diffuse enlargement of the thyroid compared with controls. Thyroid gland sections stained with HE showed that the Con mice had intact thyroid follicles and almost no infiltration of mononuclear cells in thyroid tissue. Meanwhile, HT mice had destroyed thyroid follicles and obvious inflammatory cell infiltration in thyroid tissues (Fig. 1b). Further scoring the extent of intrathyroidal inflammatory cell infiltration showed that the severity of thyroiditis in HT mice was significantly greater than that in the control group (*P* < 0.001) (Fig. 1c).

To confirm the euthyroid HT mouse model, we also analyzed the concentrations of Tg-Ab and TPO-Ab, thyroid function-related parameters in serum. In the HT mouse model on Day E4.5, the serum Tg-Ab and TPO-Ab levels were significantly higher than in control mice (165.54 ± 7.92 IU/ml versus 12.90 ± 0.34 IU/ml, *n* = 28, *P* < 0.001; 45.78 ± 3.04 IU/ml versus 22.04 ± 1.21 IU/ml, n = 28, *P* < 0.001) (Fig. 1d and e). On the other hand, serum FT3 and FT4 levels were not significantly different between mice immunized with Tg and with FA (Fig. 1f and g). There was no difference in serum TSH concentrations between the groups, indicating that the HT mice were euthyroid (Fig. 1h). Taken together, these findings suggested the successful establishment of a euthyroid HT model in mice on Day E4.5.

### Endometrium abundance of FT3, FT4, Tg-ab and TPO-ab in Euthyroid HT mice

**Fig. 2:**
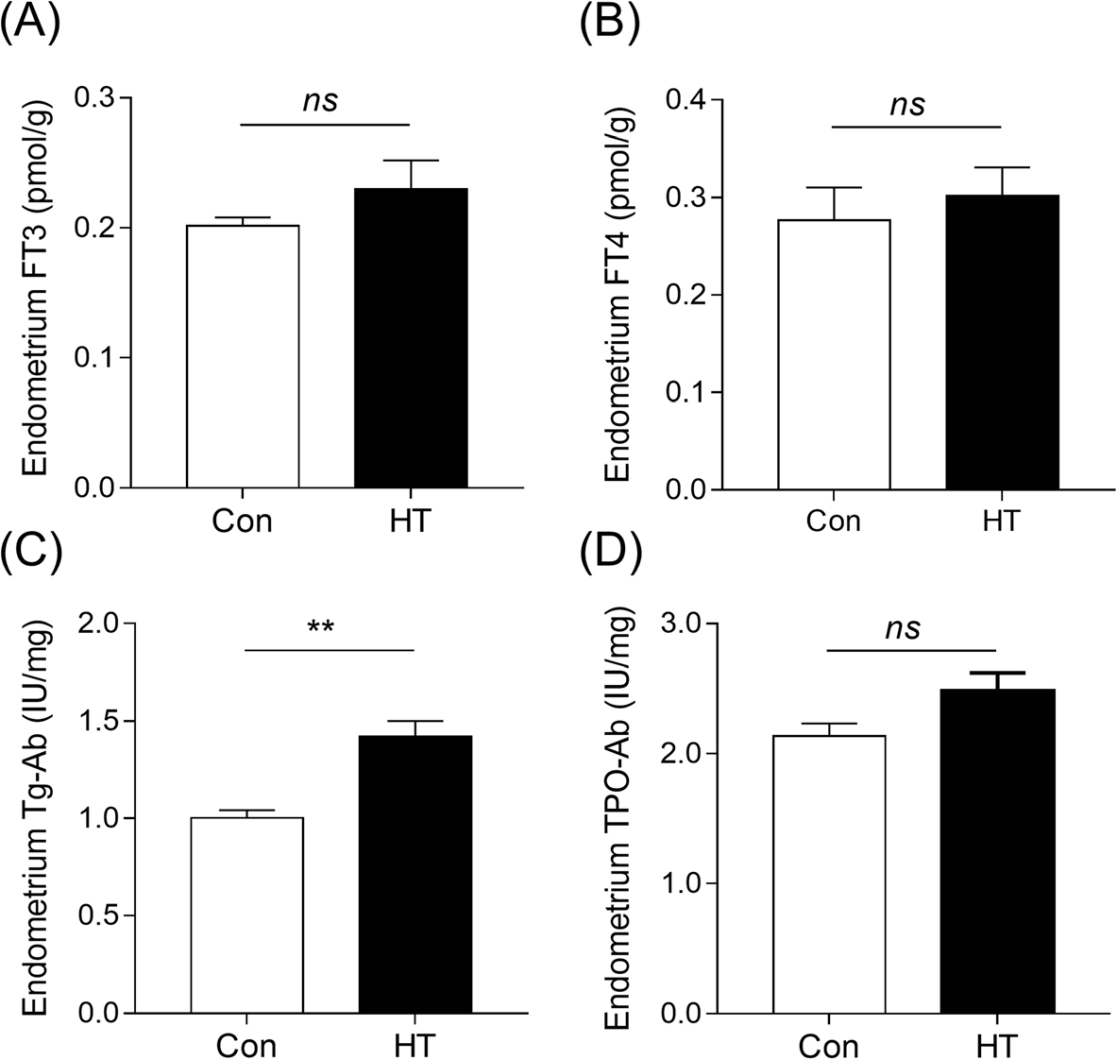
Endometrium levels of FT3, FT4, Tg-Ab and TPO-Ab in euthyroid HT mice. ECLIA was performed to detect FT3, FT4, and anti-thyroid autoantibody levels in mouse endometrial homogenate supernatant. **a** Endometrium FT3 level. **b** Endometrium FT4 level. **c** Endometrium Tg-Ab level. **d** Endometrium TPO-Ab level. Data are recorded as the mean ± SEM, *n* = 6; ns, no statistical significance; ^**^p < 0.01, vs. Con

There were no significant differences in the local endometrium levels of FT3 and FT4 examined (Fig. 2a and b). Thyroid-stimulating hormone in the local endometrium could not be detected by ELISA. The level of endometrium Tg-Ab in HT mice was significantly higher than that in controls (1.00 ± 0.04 IU/mg versus 1.42 ± 0.08 IU/mg, *n* = 6, *P* = 0.001) (Fig. 2c). Additionally, there was a tendency for a different endometrium TPO-Ab level between groups (*p* = 0.07, Fig. 2d).

### Euthyroid HT impairs embryo implantation in mice

**Fig. 3:**
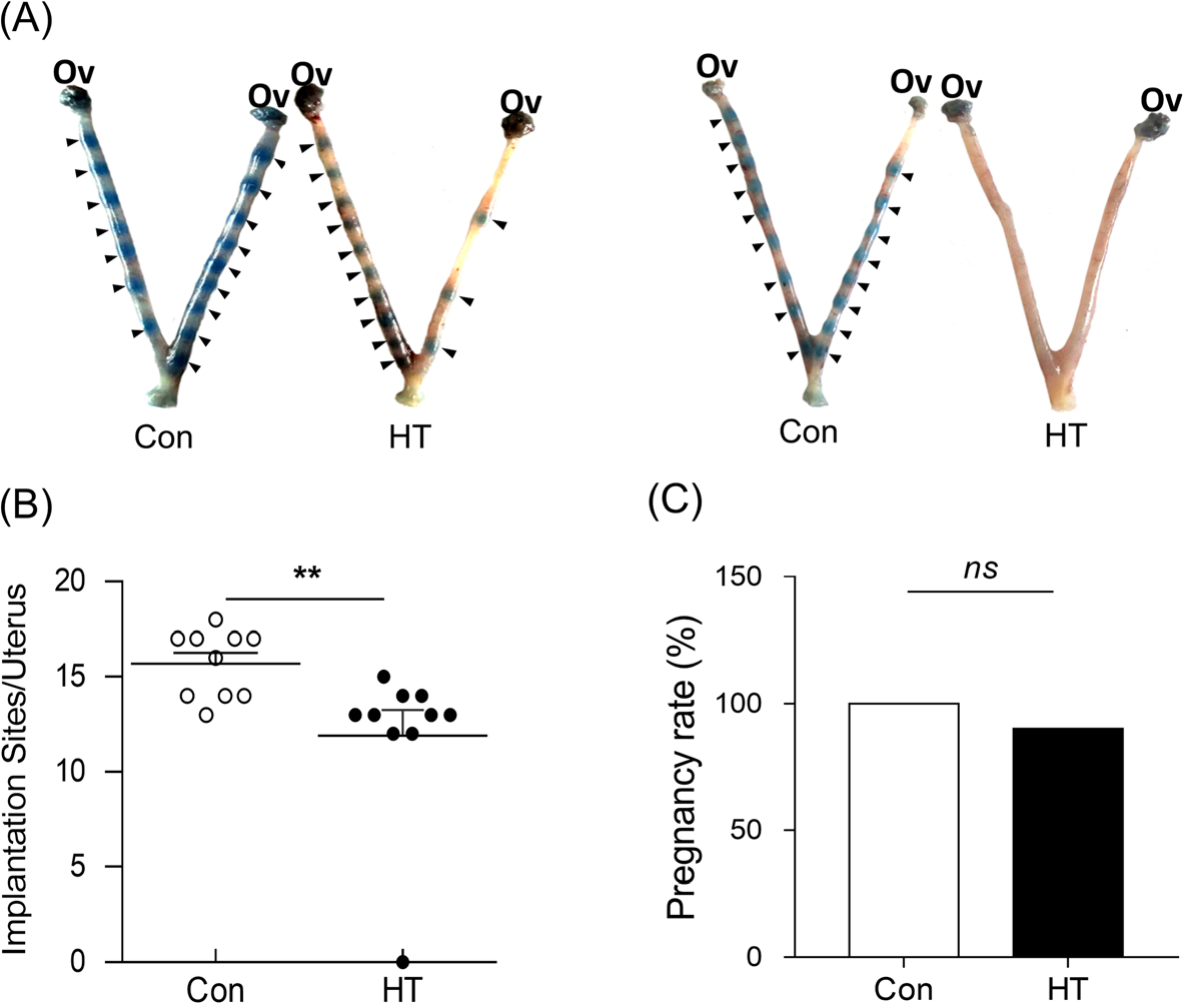
The adverse effects on embryo implantation in euthyroid HT mice. **a** Representative embryos within uteri in Con and HT mice. Arrowheads indicate viable implantation sites visualized as blue bands by uptake of Chicago Blue dye. **b** Quantitation of viable implant sites per uterus. Dots represent embryo implantation sites. Values are the mean ± SEM, *n* = 10; ns, no statistical significance; ^**^p < 0.01, vs. Con. Mann-Whitney test. **c** Statistical analyses of the prevalence of pregnancy. Ov, Ovary

We first investigated whether euthyroid HT could affect embryo implantation in mice. The macroscopic visible evidence of successful implantation is localized implantation sites that can be visualized as blue nodules by uptake of 0.1 ml 1% Chicago Blue dye solution on Day E4.5. Figure 3a shows representative uteri with embryo implantation sites (black arrows) in both groups. Control mice had dense embryonic nodules with an even distribution in uterine tissues. In contrast, HT mice had fewer embryonic nodules, with irregular distribution and larger embryonic spacing, and even no embryos in the uterine horn. Further quantitative analysis by calculating the blue nodules within each uterine horn revealed that the viable embryo implantation numbers on Day E4.5 in the HT group (13.22 ± 0.32) were fewer than those in the control group (15.70 ± 0.56; *p* **=** 0.002) (Fig. 3b). Compared to the control (100%, 10/10), the HT group showed a downward tendency in the prevalence of pregnancy (90%, 9/10) (Fig. 3c).

### Euthyroid HT induces compromised endometrial morphology in mice

As the results above indicated that euthyroid HT decreased the competence of embryo implantation, we then assessed the effect of euthyroid HT on endometrial morphology on Day E4.5 as an indication of uterine receptivity by HE and SEM observation.

### (1) Euthyroid HT causes changes in endometrial histology of mice

**Fig. 4:**
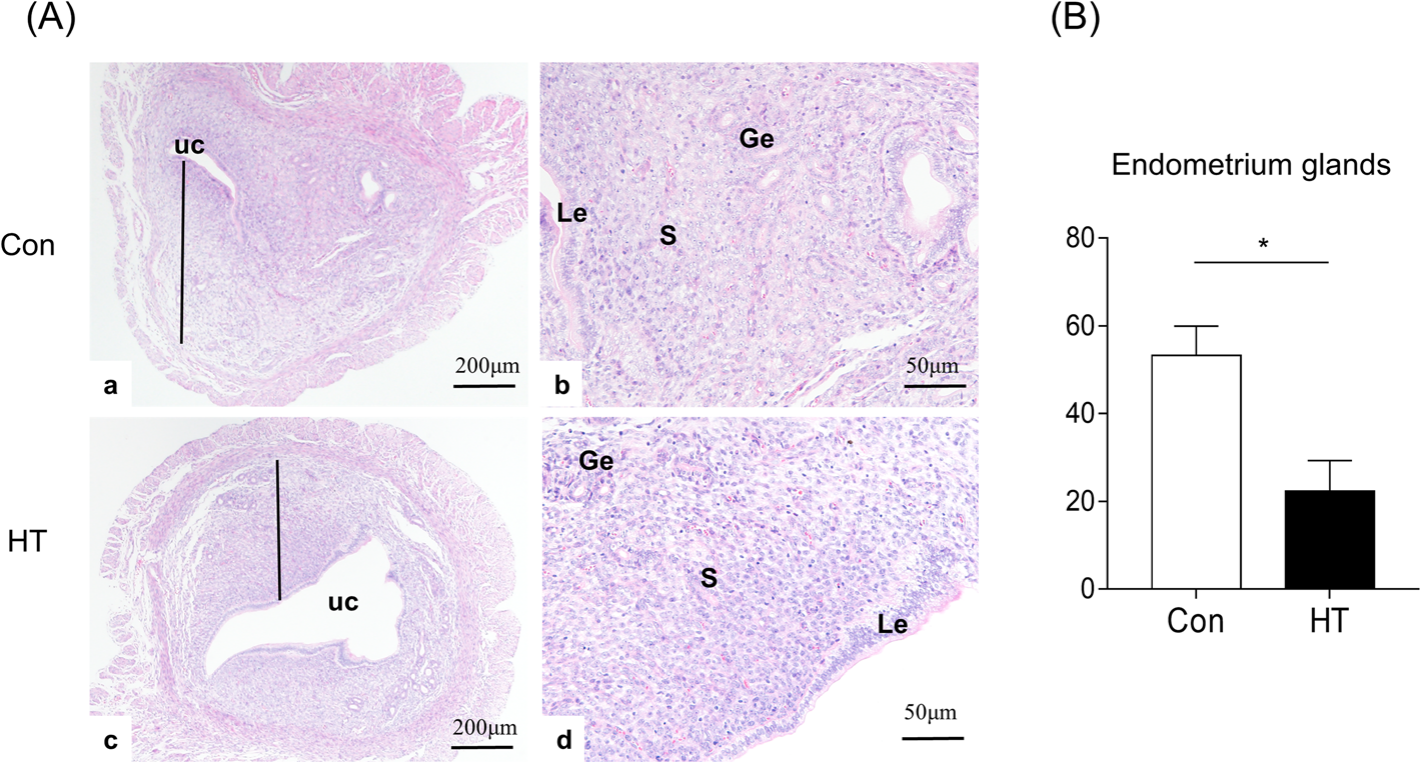
Effects of euthyroid HT on the uterine histology in mice. **A** The uterine histology detected by HE staining at magnification × 40, × 200. **B** The number of uterine glands/cross-section. UC: uterine cavity. Le: luminal glandular epithelium. S: stromal cell. Ge: glandular epithelium. The black line indicates the endometrium of the uterus. Data were recorded as the mean ± SEM, *n* = 3, ^*^p < 0.05, vs. Con

Figure 4A (a, c) shows that the endometrium was composed of the luminal epithelium, the glandular epithelium and the stroma. As depicted in Fig. 4A (b, d), the control mice exhibited normal decidualization with neat and complete luminal epithelium, a large volume of large and scattered stromal cells, and abundant and expanded glands with secretion in stroma. In contrast, HT mice revealed a failed decidualization with an open and irregular uterine lumen, incomplete development of the luminal epithelium, dense stromal cells, and fewer glands. A recent study employing a mouse model found that uterine glands play an essential role in on-time implantation and decidualization, thereby ensuring embryo viability and pregnancy success [32]. Here, quantification of the number of endometrial gland/cross section (N/CS) in the uteri revealed that euthyroid HT led to a significant reduction (53.33 ± 6.64 N/CS) compared to control uteri (22.33 ± 6.94 N/CS, *n* = 3, *p* = 0.032) (Fig. 4B).

### (2) Euthyroid HT affects the development of endometrial pinopodes in mice

**Fig. 5:**
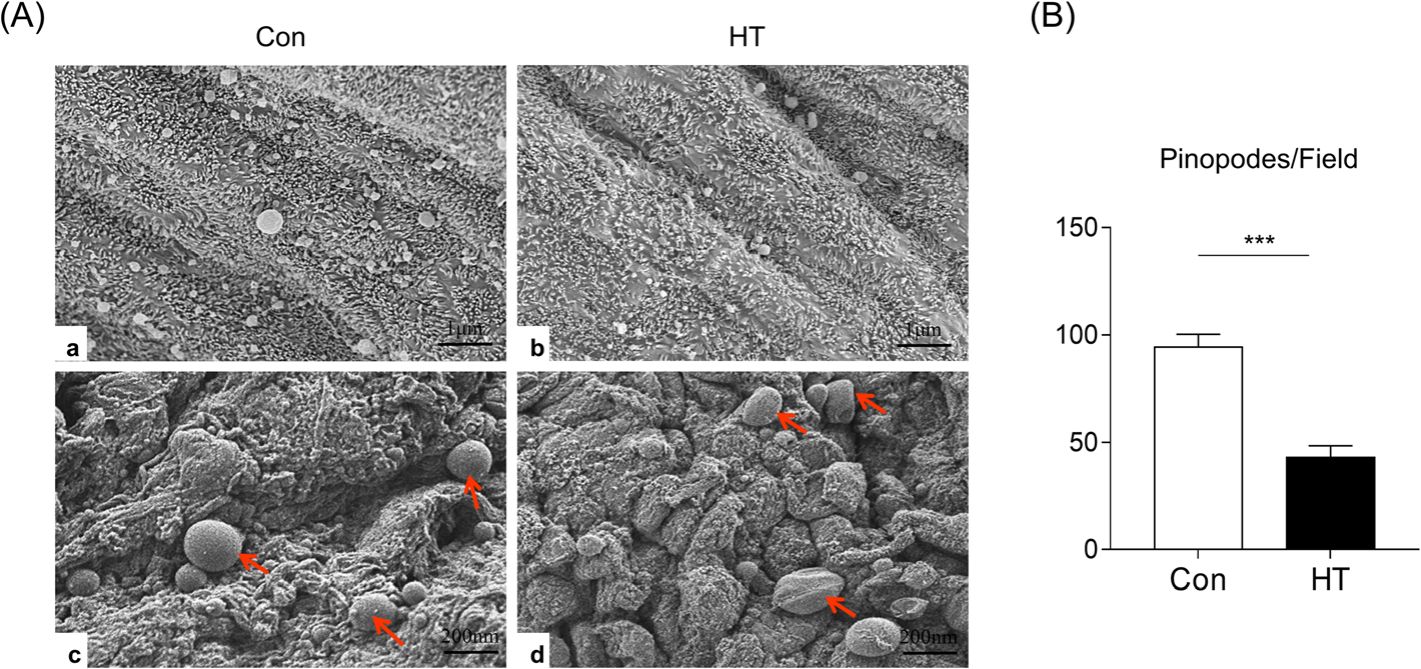
Effects of euthyroid HT on endometrial pinopodes in mice. **A** Representative SEM images of the pinopodes on the apical surface of the eutopic endometrium luminal epithelium during the window of implantation at magnification × 5000, × 10,000. Red arrows indicate endometrial pinopodes. **B** A histogram of the number of pinopodes for each microscopic field. Data were recorded as the mean ± SEM, n = 3, ^***^p < 0.001, vs. Con

We then analyzed the luminal epithelium of FA- and Tg-treated uteri for the presence of pinopodes by SEM. As shown in Fig. 5A (a, c), the endometrial surface of control mice exhibited a relatively high density of pinopodes, while the luminal surface of HT mice displayed a significant reduction in the number of pinopodes. Figure 5A (b, d) further illustrates fully developed pinopodes with characteristic dome-shaped terminal ends in Con mice. Mice with HT, however, showed abnormal pinopodes with a wrinkled or semispherical surface in the apical membrane and a sparse distribution of mature pinopodes. The statistical analysis indicated that the number of pinopodes in each microscopic field (N/EMF) in the HT group significantly decreased (43.18 ± 5.39 N/EMF) compared to the control group (94.45 ± 5.96 N/EMF, *p* < 0.001) (Fig. 5B). Taken together, these results demonstrated that HT mice had abnormal endometrial morphology.

### Euthyroid HT does not alter the serum concentration of E2 or P in mice

**Fig. 6:**
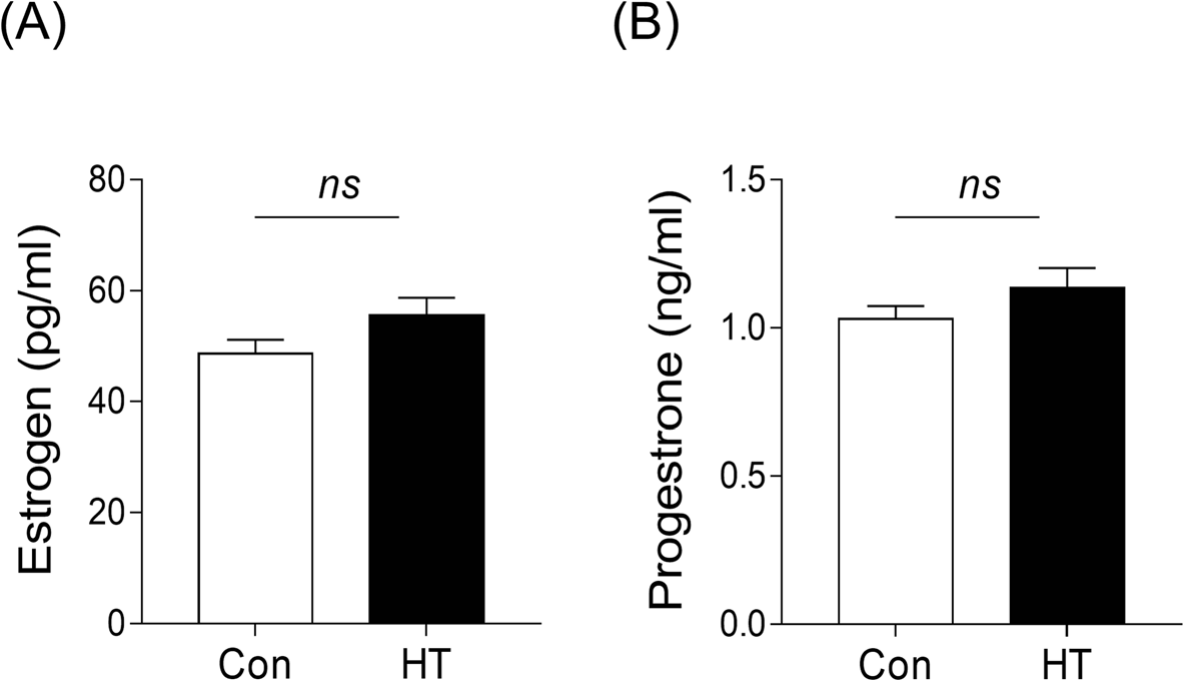
Effects of euthyroid HT on the serum E2 and P levels in mice. **a** Effects of euthyroid HT on E2 concentration in the serum. **b** Effects of euthyroid HT on the P concentration in the serum. Data are presented as the mean ± SEM; n = 28, ns, not significant, vs. Con

Establishment of endometrial receptivity is coordinately mediated by the steroids estradiol (E2) and progesterone (P). In this study, we measured the serum E2 and P levels on the morning of Day E4.5. As shown in Fig. 6a, we did not observe a significant difference in the serum concentration of E2 between the groups. The P concentrations were similar as well (Fig. 6b).

### Effects of Euthyroid HT on endometrium ERα and PR expression in mice

**Fig. 7:**
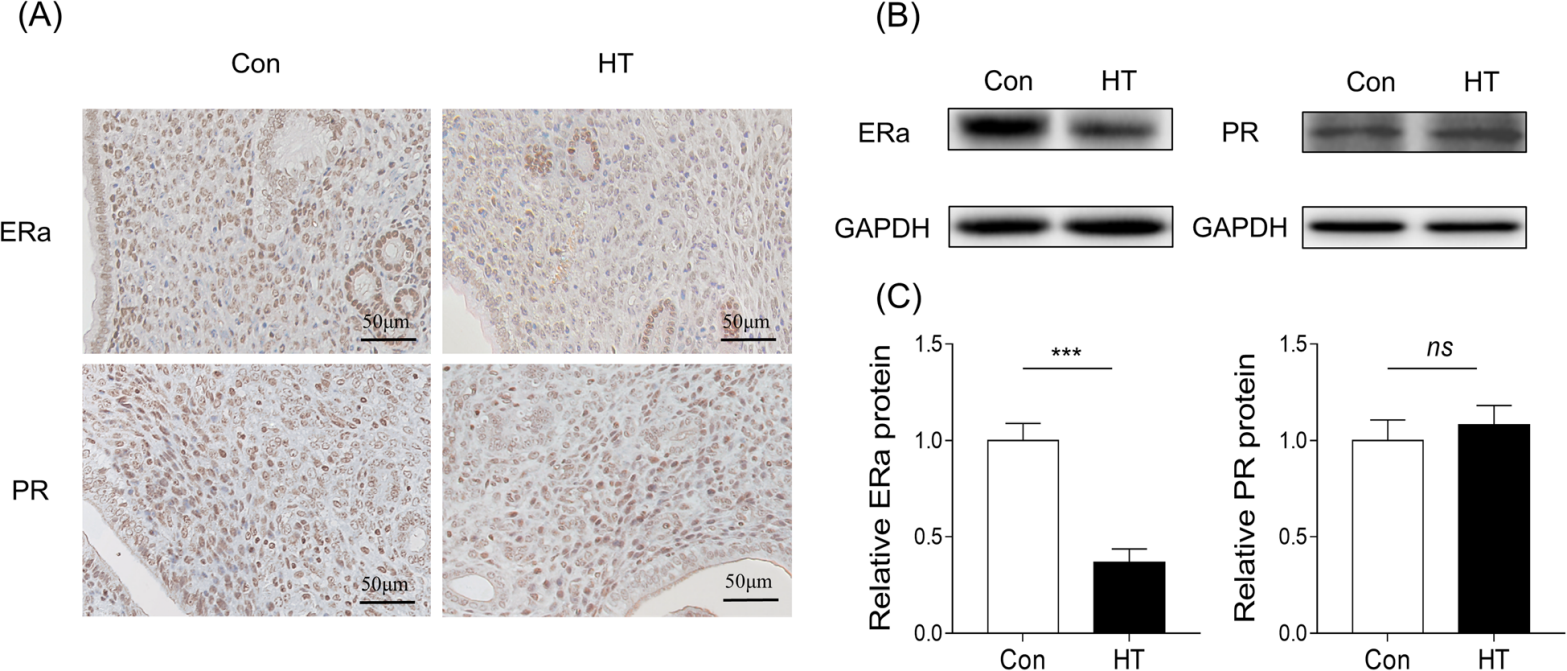
Effects of euthyroid HT on the endometrium ERα and PR expression in mouse endometrium. **a** IHC to test the effect of euthyroid HT on the protein expression of ERα and PR in pregnant mouse endometrium (*n* = 3). **b** Western blotting to test the effect of euthyroid HT on the protein expression of ERα and PR in pregnant mouse endometrium. GAPDH was blotted as a control. **c** Quantification of western blotting (*n* = 3). Each column represents the mean ± SEM; ns, not significant; ^***^p < 0.001, vs. Con

To explore the effect of euthyroid HT on endometrial receptivity, the IHC analysis showed that ERα immunostaining in the HT group was immunopositive and significantly reduced in the nucleus of the epithelial and stromal cells. Interestingly, the endometrial nuclear PR level in stromal cells was comparable between the control and HT groups (Fig. 7a). Consistent with IHC analysis, western blotting analysis revealed that, compared to control, euthyroid HT downregulated ERα protein (*p* < 0.001) in the endometrial tissues at the implantation time. There was no significant difference in the expression of PR protein between groups (*p* = 0.796) (Fig. 7b and c)

### Euthyroid HT inhibits the expression of integrin β3, ICAM-1 and Galectin-3 in mouse endometrium

**Fig. 8:**
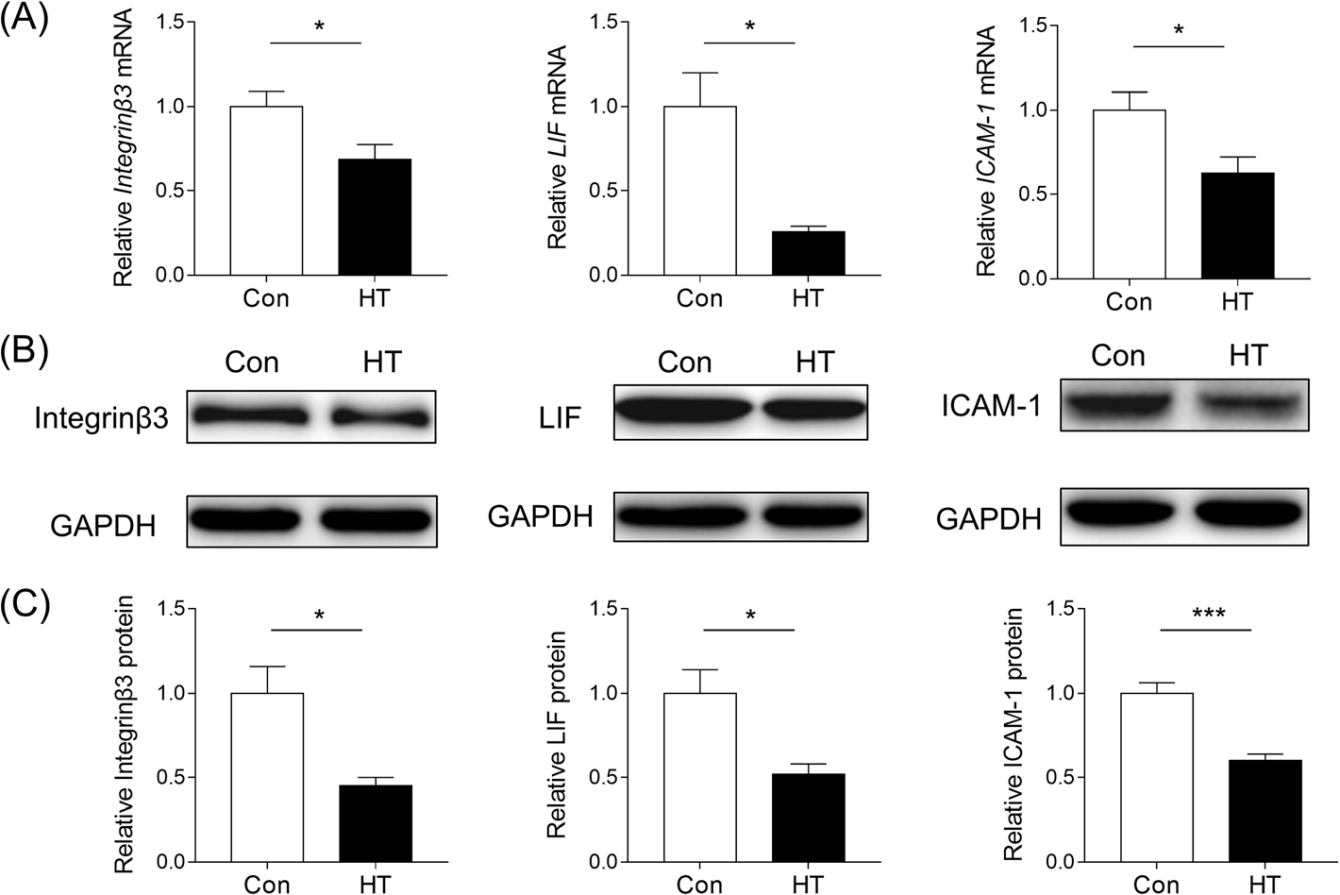
Effects of euthyroid HT on receptivity markers expression in mice. **a** The mRNA levels of integrin β3, LIF and ICAM-1 in the endometrium were measured by RT-PCR. Values are normalized to the β-actin expression level (*n* = 6). **b** Representative western blot for integrin β3, ICAM-1 and galectin-3 protein in endometrial tissue lysates from each group. GAPDH was used as a loading control (*n* = 6). **c** Quantification of western blotting. Each column represents the mean ± SEM; ^*^p < 0.05, ^***^p < 0.001, vs. Con

To further investigate the effect of euthyroid HT on endometrial receptivity in the implantation window, the expression of integrin β3, LIF and ICAM-1 was analyzed. The results of RT-PCR illustrated that the mRNA abundance of integrin β3, LIF and ICAM-1 were significantly decreased in HT mice compared to the controls (*p* = 0.031, *p* = 0.012, *p* = 0.025, respectively; Fig. 8a). The findings of western blotting supported this result, and compared with controls, abundance of integrin β3, LIF and ICAM-1 in the endometrium on Day E4.5 were downregulated in HT mice (*p* = 0.017, *p* = 0.016, *p* < 0.001, respectively; Fig. 8b and c).

## Discussion

Circulating Tg-Ab and TPO-Ab are now considered the serological hallmarks to establish a diagnosis of HT in humans. They are found in HT patients but are rare in healthy controls [1]. Previous research showed that 10.5% of repeated-implantation-failure women were positive for ATA [33]. Hashimoto thyroiditis, the most frequent AITD, is well known as the primary underlying factor leading to autoimmune hypothyroidism. Although some studies demonstrated that thyroid autoimmunity affected reproductive outcomes due to thyroid dysfunction, including implantation failure [34, 35], increasing evidence suggests that pregnancy loss is greater in women positive for Tg-Ab and TPO-Ab, regardless of the functional status of their thyroid [36, 37]. Furthermore, another study reported that euthyroid patients with high serum TPO-Ab and TG-Ab showed impaired embryo implantation [13]. Whereas some other studies reported that a comparable pregnancy rate had been observed after assisted reproductive technology in women with and without AITD in the euthyroid state, it is believed that AITD itself does not alter embryo implantation [38, 39]. To date, there is a lack of well-designed animal experiments to elucidate the effects of euthyroid HT on embryo implantation events. Tg-induced thyroiditis in susceptible mice is a classic model of HT to explore the pathogenesis and therapeutics of HT [40]. A Tg-induced euthyroid HT mouse model previously established in our laboratory was used for the study [41]. Thus, to examine the isolated effect of euthyroid HT on embryo implantation, this study utilized female NOD mice immunized with porcine Tg, resulting in pronounced diffuse enlargement of the thyroid gland, the production of intrathyroidal mononuclear cell infiltration and rising serum Tg-Ab and TPO-Ab antibodies without accompanying FT3, FT4 and TSH abnormalities in serum and the local endometrium, which defines euthyroid HT in humans. To our knowledge, our study is the first to use this model to explore the effect of HT itself on embryo implantation in mice, focusing on the possible contribution of uterine receptivity in mediating such an effect. Here, HT mice displayed decreased embryo implantation numbers on Day E4.5 compared with controls despite similar thyroid hormone concentrations between groups. These findings suggest that HT itself affected reproductive outcomes relevant to compromised embryo implantation in mice, providing preliminary evidence to support the hypothesis linking euthyroid HT to implantation failure.

In early pregnancy, a receptive endometrium is critical for successful embryo implantation [42]. Hashimoto thyroiditis is the most prevalent autoimmune disease, and other studies reported that autoimmune disease alters the endometrial receptivity, affecting implantation [16, 43]. Hence, to ascertain whether the deleterious effect of euthyroid HT on embryo implantation was associated with impaired maternal endometrial receptivity, the mouse endometrial morphology in this study was examined by HE staining and SEM. Euthyroid HT mice assessed by HE staining on Day E4.5 displayed decreased endometrial thickness, irregular and open uterine lumen, incomplete development of the luminal epithelium, and dense stromal cells. In addition, the number of endometrial glands was reduced; these glands play an essential role in embryo development and implantation, thereby ensuring pregnancy establishment and success [44]. These endometrial morphology anomalies in euthyroid HT mice may be responsible for low fertility. Pinopodes are specific morphological biomarkers for endometrial receptivity due to their spatiotemporal expression [45, 46], which suggests an “open window” period facilitating implantation [47, 48]. A previous TEM study demonstrated that pinopodes contain secretory vacuoles that extend into the lumen and that their material may provide nutrients for the embryo, favoring its attachment to the endometrium [49]. During the WOI, pinopodes absorb macromolecules and fluid from the uterine lumen and prevent movement of the cilia that is coordinated with generalized stromal edema to induce lumen closure assisting embryo attachment to the epithelium [50, 51]. According to in vitro studies, the smooth pinopode surface, which is the preferential site for blastocyst attachment, has stronger adhesion to the embryo than the microvilli surface for implantation [52]. Published data suggested that women with reduced implantation displayed few or no pinopode [53]. In the present study, the luminal surface of HT mice presented abnormal pinopodes with a wrinkled or small semispherical surface in the apical membrane and a sparse distribution of mature pinopodes, and the number of pinopodes in each unit area was significantly reduced on SEM. All these findings suggested euthyroid HT may affect luminal epithelium development, inhibiting the formation and development of pinopodes, thereby impairing endometrial receptivity and leading to embryo implantation failure.

A specialized environment in utero is essential for successful blastocyst implantation in mammalian reproduction. E2 and P regulate the growth and differentiation of reproductive tissues for implantation by specifically binding to their nuclear receptors ERα and PR to maintain the uterine environment [54–56]. Interestingly, despite the impaired implantation response, no statistical changes in serum E2 or P were detectable by ELISA in HT mice. This result is consistent with previous studies indicating that a reduced implantation rate was not related to steroid damage in ATA-positive women [13, 57]. It is interesting to speculate that endometrial receptivity for implantation is not affected in HT mice at the level of E2 or P action. Analyses of IHC and WB, however, showed significant downregulation of ERα but no difference in PR in the HT group. During the receptive window, epithelial ERα is indispensable for ceasing epithelial cell proliferation to allow embryo adhesion, indicating that epithelial ERα regulates implantation [58]. Another study demonstrated that loss of stromal ERα caused fewer pups in mice, in part due to the inability of some embryos to implant in the uterus [59]. In another ER−/− mouse model with ERα conditionally deleted from the uterine epithelial and stromal compartments showed a complete loss of decidual response, indicating ERα played an essential role in the regulation of decidualization [60]. According to the critical roles of ERα in reproduction, we hypothesized that the aberrant expression of ERα caused by euthyroid HT was related to implantation failure in the HT group.

The major factors in the establishment of uterine receptivity for implantation are determined by E2 and P combined with their nuclear receptors, as well as adhesion molecules, growth factors and cytokines [61]. To further explore the effects of HT itself on endometrial receptivity, we investigated integrin β3, LIF, and ICAM-1 expression in endometrial tissues. WB and RT-PCR analyses showed that, compared to the controls, the protein and mRNA expression levels of integrin β3, LIF and ICAM-1 were all significantly decreased in HT mice on Day E4.5. Integrin β3 promotes adhesion through cell-cell interactions and is considered a biomarker for evaluating uterine receptivity, with high levels also facilitating embryo attachment [62, 63]. In the mouse model, integrin β3 expression peaks during WOI, and if this increase is blocked, implantation is significantly inhibited with decreased embryo implantation number [64]. As one of the main and effective molecules in endometrial receptivity, LIF spatiotemporal expression in the endometrium may initiate implantation of blastocysts during WOI [65]. In a transgenic mouse homozygous for the LIF model, the blastocyst is viable but not implantable, indicating that the maternal LIF is critical for implantation [66]. ICAM-1 is a molecular marker associated with the menstrual cycle and the presence of pinopode microRNA [67]. The results of WB and RT-PCR showed that HT mice had decreased levels of integrin β3, LIF and ICAM-1. Reduced expression of integrin β3, LIF and ICAM-1 may explain the observation that euthyroid mice with HT seem to have exceptional implantation due to impaired endometrial receptivity.

The mechanisms by which HT induces impaired embryo implantation and endometrial receptivity in the euthyroid state are still unknown. On the one hand, it has been reported that the possible mechanisms of Tg-Ab and TPO-Ab related to infertility and pregnancy morbidities include that ATA induces thyroid dysfunction [68, 69]. Studies have shown that alterations of the highly regulated local activity of thyroid hormones directly interfere with embryo attachment and early implantation in hypothyroidism [6, 7]. Different pathophysiological mechanisms have been proposed that hyperthyroidism was related to infertility, since increased serum androgens, estradiol concentrations and luteinizing hormone response to gonadotropin-releasing hormone led to menstrual disturbances [70, 71]. In another study, it has been suggested that hyperthyroidism had an impact on uterine oxidative stress owing to alterations of the total superoxide dismutase, catalase and glutathione peroxidase activities in utero, thus influencing fertility [72]. In addition, the literature has indicated that local tissue thyroid hormone deficiency was noted prior to plasma depletion, suggesting thyroid hormone dysfunction in local tissue despite plasma thyroid hormones in the normal range [73]. In our study, compromised endometrial receptivity, including endometrial morphology anomalies and molecular features changes, was unlikely to be due to thyroid dysfunction because the thyroid hormones, not only in serum but also in the local endometrium, were within the normal range. Moreover, increased thyroid antibodies themselves may also be pathogenic, given that TPO-Ab specifically binds to chorionic gonadotropin receptors and placental antigens to affect fetal resorptions in an animal model [74]. A study demonstrated the presence of antigenic sites for ATA on reproductive tissue [75], and abnormal immune recognition of placental antigens and Tg by Tg-Ab has been described in mice immunized with Tg, which experienced pregnancy loss and a decrease in placental and fetal weight even if thyroid hormones are within the normal range [76]. This study displayed rising ATA levels in the local endometrial tissue of HT mice. It appears reasonable to then propose that ATA may cross-react with autoantigens expressed in the endometrium and modulate local immune responses. Further studies are needed to explore the detailed mechanisms of action of the uterine factors in the context of euthyroid HT.

## Conclusions

In conclusion, we explored the effects of HT itself on reproduction from the perspective of embryo implantation, which is a critical stage of pregnancy. Our results suggested that euthyroid HT impaired embryo implantation through induction of endometrial receptivity defects, including altered morphology and disrupted expression of ERα, integrin β3, LIF, and ICAM-1 in the endometrium. Our findings could provide a useful foundation for studying euthyroid HT in pregnancy loss. In addition, this study focused on the receptive uterus, which is one of the core factors for successful embryo implantation. Other main factors relevant to embryo implantation regulation, such as competent blastocysts, should be explored in further studies.

## Data Availability

The datasets used and/or analyzed during the current study are available from the corresponding author on reasonable request.

## Abbreviations

AITD: Autoimmune thyroid disease
ATA: Antithyroid autoantibody
cDNA: complementary DNA
CFA: Complete Freund’s adjuvant
dNTP: Deoxynucleotide triphosphate
E2: Estradiol
ECLIA: Electrochemiluminescence immunoassay
ELISA: Enzyme-linked immunosorbent assay
ERα: Estrogen receptor α
FT3: Free triiodothyronine
FT4: Free tetraiodothyronine
HE: Hematoxylin and eosin
HT: Hashimoto’s thyroiditis
ICAM-1: Cell adhesion molecule-1
IFA: Incomplete Freund’s adjuvant
IHC: Immunohistochemistry
LIF: Leukemia inhibitory factor
P: Progesterone
PBS: Phosphate-buffered saline
PMSF: Phenylmethylsulfonyl fluoride
PR: Progesterone receptor
RT-PCR: Reverse transcription-quantitative polymerase chain reaction
SDS: Sodium dodecylsulfate
SEM: Scanning electron microscopy
Tg: Thyroglobulin
Tg-Ab: Anti-thyroglobulin antibody
TH: Thyroid hormones
TPO-Ab: Anti-thyroid peroxidase antibody
TSH: Thyroid-stimulating hormone
